# Expansion and Impaired Mitochondrial Efficiency of Deep Subcutaneous Adipose Tissue in Recent-Onset Type 2 Diabetes

**DOI:** 10.1210/clinem/dgz267

**Published:** 2019-12-15

**Authors:** Kálmán Bódis, Tomas Jelenik, Jesper Lundbom, Daniel F Markgraf, Alexander Strom, Oana-Patricia Zaharia, Yanislava Karusheva, Volker Burkart, Karsten Müssig, Yuliya Kupriyanova, Meriem Ouni, Martin Wolkersdorfer, Jong-Hee Hwang, Dan Ziegler, Annette Schürmann, Michael Roden, Julia Szendroedi, GDS Study Group, A E Buyken, B Belgardt, G Geerling, H Al-Hasani, C Herder, J H Hwang, A Icks, J Kotzka, O Kuss, E Lammert, D Markgraf, K Müssig, W Rathmann, J Szendroedi, D Ziegler, M Roden

**Affiliations:** 1 Division of Endocrinology and Diabetology, Medical Faculty, Heinrich Heine University, Düsseldorf, Germany; 2 Institute for Clinical Diabetology, German Diabetes Center, Leibniz Center for Diabetes Research at Heinrich Heine University, Düsseldorf, Germany; 3 German Center for Diabetes Research (DZD), München-Neuherberg, Germany; 4 Department of Experimental Diabetology, German Institute of Human Nutrition Potsdam-Rehbrücke, Nuthetal, Germany; 5 Landesapotheke Salzburg, Salzburg, Austria

**Keywords:** Adipose tissue, humans, insulin resistance, metabolic flexibility, mitochondrial function, type 2 diabetes

## Abstract

**Context/Objective:**

Impaired adipose tissue (AT) function might induce recent-onset type 2 diabetes (T2D). Understanding AT energy metabolism could yield novel targets for the treatment of T2D.

**Design/Patients:**

Male patients with recently-diagnosed T2D and healthy male controls (CON) of similar abdominal subcutaneous AT (SAT)-thickness, fat mass, and age (n = 14 each), underwent hyperinsulinemic-euglycemic clamps with [6,6-^2^H_2_]glucose and indirect calorimetry. We assessed mitochondrial efficiency (coupling: state 3/4o; proton leak: state 4o/u) via high-resolution respirometry in superficial (SSAT) and deep (DSAT) SAT-biopsies, hepatocellular lipids (HCL) and fat mass by proton-magnetic-resonance-spectroscopy and -imaging.

**Results:**

T2D patients (known diabetes duration: 2.5 [0.1; 5.0] years) had 43%, 44%, and 63% lower muscle insulin sensitivity (IS), metabolic flexibility (*P* < 0.01) and AT IS (*P* < 0.05), 73% and 31% higher HCL (*P* < 0.05), and DSAT-thickness (*P* < 0.001), but similar hepatic IS compared with CON. Mitochondrial efficiency was ~22% lower in SSAT and DSAT of T2D patients (*P* < 0.001) and ~8% lower in SSAT vs DSAT (*P* < 0.05). In both fat depots, mitochondrial coupling correlated positively with muscle IS and metabolic flexibility (*r* ≥ 0.40; *P* < 0.05), proton leak correlated positively (*r* ≥ 0.51; *P* < 0.01) and oxidative capacity negatively (*r* ≤ −0.47; *P* < 0.05) with fasting free fatty acids (FFA). Metabolic flexibility correlated positively with SAT-oxidative capacity (*r* ≥ 0.48; *P* < 0.05) and negatively with DSAT-thickness (*r* = −0.48; *P* < 0.05). DSAT-thickness correlated negatively with mitochondrial coupling in both depots (*r* ≤ −0.50; *P* < 0.01) and muscle IS (*r* = −0.59; *P* < 0.01), positively with FFA during clamp (*r* = 0.63; *P* < 0.001) and HCL (*r* = 0.49; *P* < 0.01).

**Conclusions:**

Impaired mitochondrial function, insulin resistance, and DSAT expansion are AT abnormalities in recent-onset T2D that might promote whole-body insulin resistance and increased substrate flux to the liver.

Type 2 diabetes (T2D) is strongly related to weight gain and accumulation of excess fat within the liver. Abnormal adipose tissue (AT) function, which is induced by chronic overnutrition or is a result of lipodystrophy, promotes insulin resistance and ectopic lipid accumulation within skeletal muscle, liver, and heart (1). This likely contributes to the onset of T2D (2–4).

Weight loss and particularly the reduction of body fat content (as a result of lifestyle intervention or bariatric surgery), have been shown to achieve normalization of blood glucose levels in people with recently-diagnosed T2D (5). The major determinants of this response to the remission strategies are the extent of body fat loss, duration of T2D, and residual beta cell function (6). While the restoration of normal glucose tolerance has been allocated to beta cell recovery, the metabolic disturbance of AT in recent-onset, yet reversible, T2D has gained growing interest.

Glucose homeostasis is maintained by the integrated adaptation of substrate flux to prevailing metabolic conditions in skeletal muscle, white AT, and the liver (7). Metabolic flexibility refers to the ability of insulin-sensitive individuals to switch from lipid to carbohydrate oxidation in response to insulin (8, 9). Impairment of metabolic flexibility strongly relates to the reduction of mitochondrial plasticity in the skeletal muscle, but might also involve AT mitochondrial function (10, 11). Adipocyte insulin signaling and insulin-stimulated glucose uptake depend on mitochondrial efficiency (12, 13). Accordingly, the expression of proteins regulating mitochondrial function in abdominal subcutaneous AT (SAT) is lower in insulin-resistant compared with insulin-sensitive humans (14). In mice, impaired mitochondrial function in white AT directs the flux of lipid metabolites and lactate to the skeletal muscle and the liver, which results in ectopic lipid deposition and insulin resistance (15). Similarly, the discoordination of metabolic adaptation and energy metabolism in AT might also contribute to the onset of T2D and hepatic steatosis in humans who are at risk for impaired glucose homeostasis (16).

Human abdominal AT consists of SAT and visceral AT (VAT). In lean humans, SAT accounts for 80% to 90% of total body fat mass (17) and is divided by Scarpa’s fascia into a superficial (SSAT) and a deep layer (DSAT). SSAT volume associates negatively with glycated hemoglobin (HbA_1c_) in T2D (18), whereas DSAT volume-similar to VAT-relates to hepatic and whole-body insulin resistance (19, 20).

In general, DSAT expression profile was an intermediary to both SSAT and VAT, but reflected more of the VAT expression profile (21). Thus, the intracellular pool of glucose transporter type 4 proteins was lower in DSAT (21). Accordingly, lower protein expression of glucose transporter type 4 was found in DSAT as compared with SSAT, which likely reflects lower glucose uptake in DSAT (22). Moreover, DSAT showed a higher ratio of saturated than monounsaturated fatty acids (23). Thus, DSAT expansion could have adverse metabolic consequences, but its relation to tissue-specific insulin sensitivity has not been yet analyzed. Only a few studies have assessed mitochondrial function via direct measurements of respiration in SAT, yielding controversial results. While there is evidence for metabolic differences between both compartments, their mitochondrial function has not been assessed yet.

Our objective was to assess mitochondrial function in SSAT and DSAT separately and its relation to tissue-specific insulin sensitivity of the skeletal muscle, liver, and AT as well as steatosis and metabolic flexibility in recently-diagnosed T2D patients independently of total fat mass. We hypothesized that impaired mitochondrial function in DSAT associates with tissue-specific insulin resistance, impaired glucose homeostasis, lower metabolic flexibility, ectopic lipid deposition, and expansion of DSAT thickness.

## Materials and Methods

### Participants

From the German Diabetes Study (GDS), 14 male patients with T2D and 14 men with normal glucose tolerance (CON) were recruited. They were matched for sex, age, body mass index (BMI), total body fat mass and whole SAT (WSAT) thickness. The GDS is a prospective observational study, which investigates the natural course of recently-diagnosed diabetes and the development of diabetes-associated complications compared with a cohort with normal glucose tolerance. The study design and cohort profile of GDS have been described before (24). All participants gave written informed consent before inclusion in the study (ClinicalTrial.gov registration no: NCT01055093), which was performed according to the Declaration of Helsinki and approved by the ethics board of Heinrich Heine University, Düsseldorf, Germany. In order to be eligible for the present study, the participants needed to have been diagnosed with T2D for a period of less than 7 years, to have a minimium BMI of 25 kg/m^2^ and to be between the ages of 35 to 70 years. Specific exclusion criteria were the history of acute or chronic diseases including cancer, medication affecting the immune system, treatment with insulin or thiazolidinediones, and a HbA_1c_ level above 9.5% (80 mmol/mol) or a positive family history for diabetes in CON. The participating patients with T2D were prescribed with either lifestyle modification only (n = 2), metformin only (n = 9), sulfonylurea only (n = 1), metformin plus sulfonylurea (n = 1) or a dipeptidyl-peptidase-4-inhibitor plus metformin (n = 1). Those patients on oral glucose-lowering medication withdrew their treatment for at least 3 days before all measurements (24) and none of the recruited patients received thiazolidinediones. Therefore, a confounding effect of diabetes medication on mitochondrial function was unlikely (25). Three CON and 9 patients with T2D were treated with antihypertensive medication, 6 T2D patients received lipid-lowering medication and 3 T2D patients were treated with antiplatelet drugs.

### Study protocol

For 3 days prior to each visit, participants refrained from physical activity and alcohol ingestion as well as adhered to a balanced isocaloric diet. For all visits, the participants arrived at the Clinical Research Center in the morning after 10 hours of overnight fasting. On the first day, each participant provided their medical history and underwent bioimpedance analysis and ultrasound imaging as well as physical examination and laboratory tests. On the second day, proton-based magnetic resonance spectroscopy (^1^H MRS) and magnetic resonance imaging (MRI) measurements were followed by ultrasound imaging and SAT biopsies. On the third day, participants underwent a hyperinsulinemic-euglycemic clamp test with isotope dilution technique for an assessment of tissue-specific insulin sensitivity and with indirect calorimetry for an assessment of mitochondrial flexibility. All CON underwent a standardized 75-g oral glucose-tolerance test (OGTT) (Accu-Chek Dextro O.G-T., Roche, Basel, Switzerland) with blood sampling at time points of 5, 30, 60, and 120 minutes to assess glucose tolerance and to exclude persons with impaired glucose tolerance (24). Dysglycemia was categorized according to the current internationally accepted criteria (26).

### Hyperinsulinemic-euglycemic clamp test

The clamp test was performed according to the Botnia protocol with an intravenous glucose-tolerance test and a subsequent clamp test (24). The latter was started with a priming dose of 10 mIU/(body weight [kg]*min) for 10 minutes, followed by a continuous infusion of short-acting human insulin (Insuman Rapid, Sanofi-Aventis, Frankfurt am Main, Germany) 1.5 mU/(body weight [kg]*min) for 3 hours to assess muscle and hepatic insulin sensitivity (24). Combined with continuous infusion of deuterated glucose (D-[6,6-^2^H_2_]glucose), the clamp yields insulin-stimulated rates of glucose disappearance as a measure of skeletal muscle insulin sensitivity (16). Hepatic insulin sensitivity was measured as difference between basal and insulin-suppressed endogenous glucose production (∆EGP) using a continuous infusion of D-[6,6-^2^H_2_]glucose (24). Insulin sensitivity of AT was assessed by suppression of the plasma concentrations of endogenous free fatty acids (FFA) during the clamp test expressed as percent of FFA suppression from baseline and calculated as 1 – (average FFA during steady-state / baseline FFA) (27). Lower insulin-mediated suppression of lipolysis reflects insulin resistance of AT in vivo (16).

### Whole-body substrate oxidation

The total energy expenditure is measured in vivo noninvasively via open circuit indirect calorimetry to assess glucose and lipid oxidation by oxygen uptake (VO_2_) and the carbon dioxide output (VCO_2_) under resting conditions in order to calculate the respiratory quotient (RQ). These methods have been described elsewhere in detail (24). To quantify metabolic flexibility of the substrate oxidation the measurement is repeated at the steady state period, generally during the last 30 minutes of the hyperinsulinemic-euglycemic clamp (28). The difference between basal and insulin-stimulated RQ is an estimate of metabolic flexibility.

### Laboratory analyses

Plasma glucose, insulin, FFA, HbA_1c_, high-density lipoprotein (HDL) cholesterol, low-density lipoprotein (LDL) cholesterol, triglycerides (TG), and high-sensitivity C-reactive protein (hsCRP) were measured in the fasted state as described (24). The AT insulin resistance index (Adipo IR) was calculated as the product of the fasting plasma FFA concentration (mmol/L) and fasting plasma insulin concentration (pmol/L) and reflects AT insulin resistance (16, 29). Higher systemic FFA appearance during fasting reflects impaired insulin-mediated suppression of lipolysis and thereby determines insulin resistance of AT. Accordingly, the Adipo IR was also calculated as the product of mean plasma insulin and mean plasma FFA during the steady state of the clamp, which reflects AT insulin resistance in the postabsorptive state (16, 30).

### ^1^H-based magnetic resonance

Localized proton spectra were obtained with a 3-T whole-body magnet (Philips X-series Achieva, Best, The Netherlands) from the liver. Hepatocellular lipid content (HCL) and whole-body fat mass were measured as described (24). In 19 participants, the thickness of SAT layers was measured by single-slice MRI acquired from the same defined paraumbilical position at the level of L4 to validate SSAT, DSAT, and WSAT thickness assessed from ultrasound images.

### Ultrasound imaging of WSAT

The thickness of SSAT and DSAT was measured via 2-dimensional ultrasound imaging (Logiq S8 from GE Healthcare, Munich, Germany) using a 12 MHz linear array probe on the first and second day of visit. Measurements were performed on both sides of the paraumbilical region 5 times repetitively to assess the thickness of WSAT layers as described elsewhere (31). The final SSAT and DSAT thickness was computed as the mean of the 5 measurements from both sides and both days. The thickness of WSAT layers was validated by single-slice MRI. In comparison between ultrasound imaging and MRI data, the thickness of both SAT layers measured by the 2 methods displayed a strong positive correlation (*r =* 0.98 for SSAT), (*r =* 0.99 for DSAT), and (*r =* 0.99 for WSAT) (all *P* < 0.001). Thus, this confirms the agreement between the 2 methods.

### Adipose tissue biopsies

Ultrasound-guided biopsies from SSAT and DSAT were performed in the paraumbilical region above and beneath the fascia Scarpa under visual control at the level of rectus abdominis muscle by needle suction technique after administration of local anesthesia (5–10 mL of 1% lidocaine, B. Braun, Melsungen, Germany) (24). Video loops were recorded to document the correct location at each biopsy.

### High-resolution respirometry

High-resolution respirometry allows direct assessment of tissue-specific mitochondrial function, including mitochondrial oxidative capacity and efficiency. Ex vivo analysis of mitochondrial oxidative capacity was performed in freshly digitonin-permeabilized DSAT and SSAT biopsy samples in a 2-chamber oxygraph (OROBOROS Instruments, Innsbruck, Austria) using a similar protocol as described before for skeletal muscle (32). Maximal fatty acid oxidative capacity (state 3) was measured in the presence of octanoyl-carnitine (50 µmol/L), ADP (1.0 mmol/L), glutamate (10.0 mmol/L) and succinate (10.0 mmol/L). Cytochrome C (10 µmol/L) was added to test the integrity of the outer mitochondrial membrane. Respiration due to proton leak and not coupled to adenosine triphosphate (ATP) synthesis (state 4o) was measured after addition of oligomycin. Finally, maximal uncoupled respiration capacity of the electron transport chain (state u) was assessed by incremental titration with carbonyl cyanide p-[trifluoromethoxyl]-phenyl-hydrozone (fccp) (0.1 mmol/L per step). Oxygen consumption was corrected for AT wet weight (2–4 mg) and given as oxygen flux expressed as pmol/mg/s. The respiratory control ratio and the leak control ratio as well as the markers of mitochondrial coupling and proton leak, were calculated as the ratios of state 3/state 4o and state 4o/state u respiration, respectively. A high respiratory control ratio and low leak control ratio indicate tight coupling and low proton leak of mitochondrial function, which characterize high mitochondrial efficiency. Mitochondrial content was estimated from citrate synthase activity (CSA) (33–35).

### Statistical analyses

Results are given as median [first and third quartiles] or mean ± standard error of the mean (SEM). Variables were compared using Mann-Whitney U test and analysis of covariance (ANCOVA) model adjusted for BMI, age, body fat, and medication as indicated and presented for unpaired samples to determine differences between groups. Comparisons between abdominal SAT layers were performed using a paired Student *t*-test because these comparisons were made within the same individuum. Relationships between variables were investigated using partial Spearman rank correlation analyses adjusted for BMI, age, and body fat as indicated and presented. The standardized mean difference (Cohen’s d) was used for power analyses, because preliminary estimates for mean and SEM of mitochondrial function in SAT were not available from the literature. Based on the 2-sample-2-sided *t*-test, the power calculation showed that a standardized mean difference of 1.25 (for a large effect size) can be detected in a sample size of n = 14 per group with a power of 90%. As our experiments showed later, the effect size was even larger. For example, we found a Cohen’s d of 1.4 for the respiratory control ratio. All statistical tests were 2-sided and a *P* value less than 5% was accepted to indicate significant differences. All statistical analyses were performed using SPSS for Windows 23.0 (SPSS Inc., Chicago, IL). All graphs were generated using GraphPad Prism, Version 7.01 (GraphPad Software Inc., La Jolla, CA).

## Results

### Participant characteristics

Compared with CON, the T2D patients had 37% higher fasting glucose, 2.4-fold higher TG levels, 23% higher HbA_1c_, and 22% lower HDL cholesterol levels; however, both groups were similar in age, BMI, hsCRP, and total and LDL cholesterol (Table 1).

**Table 1.** Characteristics of recently-diagnosed type 2 diabetes patients and individuals with normal glucose tolerance

Variable	CON	T2D	*P*
Male / female, n	14 / 0	14 / 0	-
Diabetes duration, years	0	2.5 [0.1; 5.0]	-
Age, years	56 [46;60]	52 [46; 57]	0.847
BMI, kg/m^2^	30 [29; 34]	32 [29; 34]	0.635
Fasting blood glucose, mmol/L	4.5 [4.3; 4.7]	7.1 [5.6; 9.6]	**<0.001**
HbA_1c_,%	5.4 [5.1; 5.5]	7.0 [6.1; 7.8]	**<0.001**
HbA_1c_, mmol/mol	(35 [33; 37])	(53 [43; 61])	**<0.001**
Triglycerides, mg/dL	81 [64; 120]	196 [135; 235]	**<0.001**
Total cholesterol, mg/dL	225 [181; 246]	207 [173; 232]	0.408
HDL cholesterol, mg/dL	54 [49; 77]	42 [35; 53]	**<0.01**
LDL cholesterol, mg/dL	148 [116; 173]	142 [107; 162]	0.475
hsCRP, mg/dL	0.13 [0.06; 0.21]	0.21 [0.10; 0.35]	0.394

Data are shown as median [first; third quartile], *P* values were computed via 2-tailed Mann-Whitney U test. HDL, LDL, and hsCRP were analyzed in fasted state. All variables were assessed in n = 14 T2D and n = 14 CON participants. Abbreviations: BMI, body mass index; CON, controls (individuals with normal glucose tolerance); HbA1c, glycated hemoglobin; HDL, high-density lipoprotein; hsCRP, high-sensitivity C-reactive protein; LDL, low-density lipoprotein; T2D, type 2 diabetes.

### Fat distribution

**Figure 1.**
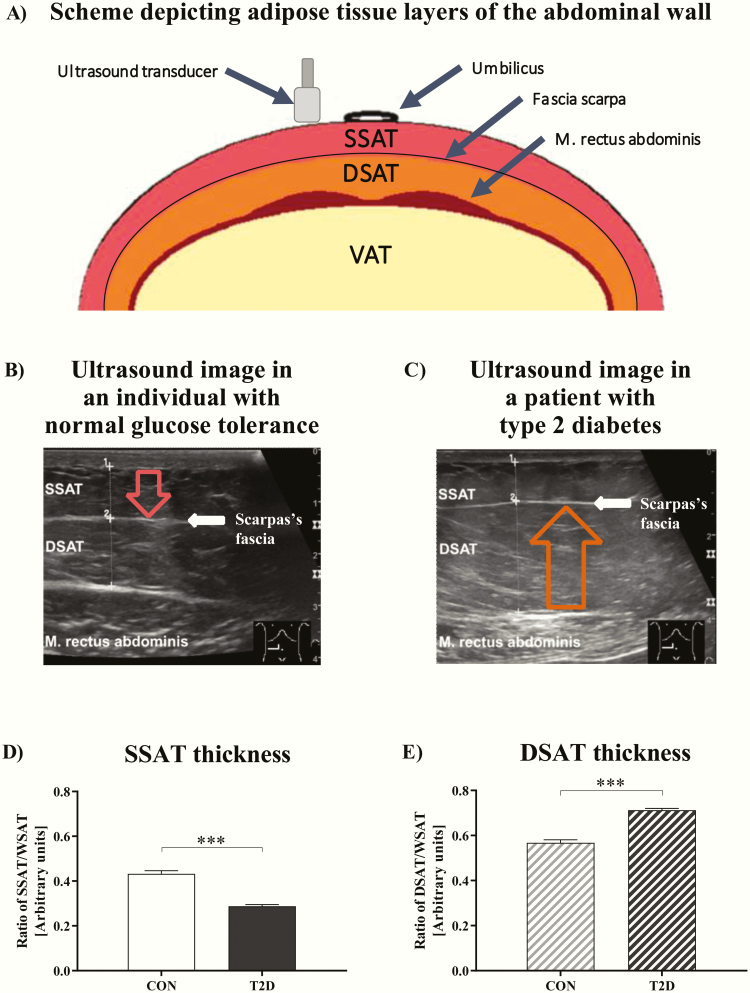
**Subcutaneous adipose tissue layers of the abdominal wall. (A)** Scheme depicting all adipose tissue layers of the abdominal wall from skin to the intestine: visceral adipose tissue (VAT), whole subcutaneous adipose tissue (WSAT) composed of superficial (SSAT) and deep subcutaneous layers (DSAT). (**B)** Ultrasound image at the level of musculus rectus abdominis showing SSAT, DSAT, and the Scarpa fascia (white line between SSAT and DSAT) dividing both adipose tissue depots in an individual with normal glucose tolerance (CON) and **(C)** a type 2 diabetes patient (T2D). The red arrow indicates the increase of SSAT thickness in CON and the orange arrow indicates the increase of DSAT thickness in T2D. **(D)** Ratio of SSAT/WSAT and **(E)** DSAT/WSAT thickness. Data are shown as mean ± SEM. ***P < 0.001, data were compared by 2-tailed Mann-Whitney U test. All variables were assessed in n = 14 T2D patients and n = 14 CON.

Both groups had similar waist circumference, total body fat mass and WSAT thickness (Table 2). Moreover, body fat mass, which was assessed by bioimpedance analysis, and the total volume of WSAT and VAT, which were measured by MRI, were comparable in both T2D and CON (Table 2). SSAT thickness was lower than DSAT thickness in both T2D (*P* < 0.001) and CON (*P* < 0.05). Despite similar total WSAT thickness (Table 2), T2D patients had lower SSAT/WSAT ratios and consequently higher DSAT/WSAT ratios than CON (Fig. 1A, 1B, 1C, 1D, and 1E). These differences were also present without normalizing for WSAT thickness (Table 2) and even after adjustments for participants’ medications. Liver fat content was 73% higher in T2D patients compared with CON (Table 2). Of note, 8 T2D patients and 4 CON met the criteria for hepatic steatosis, defined as HCL > 5.6%.

**Table 2.** Fat distribution in recently-diagnosed type 2 diabetes patients and individuals with normal glucose tolerance

Variable	CON	T2D	*P*
Waist circumference, cm	104 [97; 112]	107 [100; 113]	0.475
Body fat, %	27 [21; 37]	26 [23; 36]	0.504
Lean body weight, kg	68 [53; 80]	73 [59; 78]	0.812
SSAT thickness, cm	1.1 [0.9; 1.4]	0.8 [0.6; 1.0]	**<0.05**
DSAT thickness, cm	1.3 [1.2; 1.8]	1.9 [1.7; 2.5]	**<0.05**
WSAT thickness, cm	2.4 [2.1; 3.2]	2.7 [2.4; 3.5]	0.427
WSAT volume, cm^3§^	25 569 [16 581; 30 777]	21 344 [16 804; 28 373]	0.586
VAT volume, cm^3§^	4055 [2481; 4386]	5417 [4513; 5851]	**<0.05**
WSAT & VAT volume, cm^3§^	54 769 [31 877; 65 621]	47 268 [37 845; 61 900]	0.894
HCL, % of water signal	2.5 [1.0; 8.1]	9.2 [5.2; 20.0]	**<0.05**

Data are shown as median [first; third quartile], *P* values were computed via 2-tailed Mann-Whitney U test. Body fat and lean body weight was assessed by bioimpedance analysis. The abdominal adipose tissue tickness was assessed by ultrasound and the adipose tissue volume by magnetic-resonance-imaging. Abbreviations: CON, controls (individuals with normal glucose tolerance); DSAT, deep subcutaneous adipose tissue; HCL, hepatocellular lipid content; SSAT, superficial subcutaneous adipose tissue; T2D, type 2 diabetes; VAT, visceral adipose tissue; WSAT, whole subcutaneous adipose tissue.

§Two participants per group had no analyses of fat volume, because the measurements were not usable due to metallic implants. All other variables were assessed in n = 14 T2D and n = 14 CON participants.

### Tissue-specific insulin sensitivity

**Figure 2.**
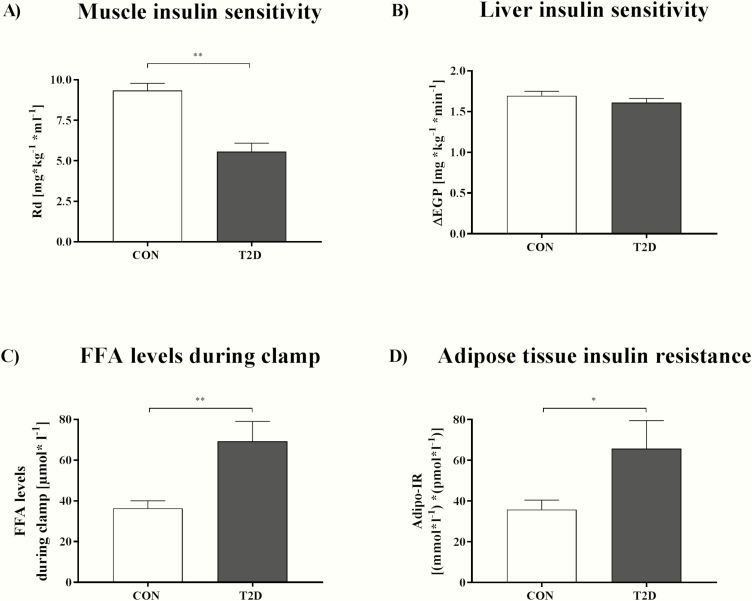
Muscle **(A)** and liver insulin sensitivity **(B)**, free fatty acid (FFA) levels during clamp **(C)** as a basis to assess adipose tissue insulin resistance **(D)**. Individuals with normal glucose tolerance (CON), rate of disappearance (Rd) for muscle insulin sensitivity, type 2 diabetes patient (T2D). The adipose tissue insulin resistance index (Adipo IR) was calculated as the product of the plasma FFA and insulin levels during the clamp and reflects adipose tissue insulin resistance. Hepatic insulin sensitivity was assessed by the difference between basal and insulin-suppressed endogenous glucose production (∆EGP). Data are shown as mean ± SEM. *P < 0.05, **P < 0.01, data were compared by 2-tailed Mann-Whitney U test. All variables were assessed in n = 14 T2D patients and n = 14 CON.

Compared with CON, T2D patients had 43% lower muscle insulin sensitivity (Fig. 2A), but similar hepatic insulin sensitivity (Fig. 2B). As detected by clamp analysis, T2D patients had 37% higher FFA during hyperinsulinemia, reflecting AT insulin resistance (Table 3, Fig. 2C). Accordingly, Adipo IR during fasting and during clamp were respectively 29% and 37% higher in T2D compared with CON (Table 3, Fig. 2D). T2D patients showed 9% lower AT insulin sensitivity than CON, as assessed by suppression of FFA during the clamp compared with baseline (Table 3).

**Table 3.** Adipose tissue insulin sensitivity in recently-diagnosed type 2 diabetes patients and individuals with normal glucose tolerance

Variable	CON	T2D	*P*
Fasting plasma insulin, pmol/L	50 [39; 87]	77 [61; 132]	0.122
Fasting plasma FFA, µmol/L	480 [376; 576]	497 [420; 710]	0.511
Clamp plasma insulin, pmol/L	972 [845; 1083]	788 [674; 1046]	0.198
Clamp plasma FFA, µmol/L^§^	34 [24; 46]	54 [43; 97]	**<0.01**
**Indices of adipose tissue insulin resistance and sensitivity**			
Adipo IR basal [(mmol/L) *(pmol/L)]	24 [16; 35]	34 [29; 53]	**<0.05**
Adipo IR clamp [(mmol/L) *(pmol/L)]	32 [22; 47]	51 [32; 82]	**<0.05**
FFA suppression, % from fasting^§^	93 [87; 94]	85 [81; 89]	**<0.05**

Data are shown as median [first; third quartile], *P* values were computed via 2-tailed Mann-Whitney U test and ANCOVA adjusted for age, BMI, and total body fat. The Adipo IR was calculated as the product of the fasting plasma FFA and insulin levels as well as the product of the plasma FFA and insulin levels during the clamp and reflects adipose tissue insulin resistance. Insulin sensitivity of adipose tissue was assessed by suppression of the plasma concentrations of FFA during the clamp expressed as percent of FFA suppression from baseline and calculated as 1 – (average FFA during steady-state / baseline FFA). All variables were assessed in n = 14 T2D and n = 14 CON participants. Abbreviations: Adipo IR, adipose tissue insulin resistance index; CON, controls (individuals with normal glucose tolerance); FFA, free fatty acids; T2D, type 2 diabetes. §Results are still significant after adjustment for age BMI and body fat.

### Whole-body substrate oxidation

**Figure 3.**
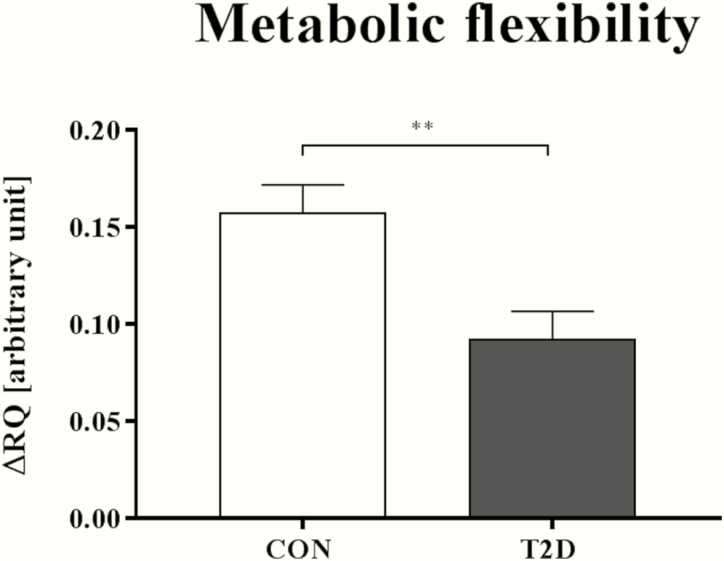
**Metabolic flexibility** Data are shown as mean ± SEM. ** *P* < 0.01, data are compared by 2-tailed Mann-Whitney U test. Difference between basal and insulin-stimulated respiratory quotient (∆RQ) to assess metabolic flexibility, individuals with normal glucose tolerance (CON) and with type 2 diabetes (T2D). In 1 participant of the control group, metabolic flexibility was not assessable, because the insulin-stimulated measurement of respiratory quotient was not performed due to technical problems. Thus, variables were assessed in n = 14 T2D patients and n = 13 CON.

Compared with CON, T2D patients had similar RQ under fasting conditions (0.79 [0.76; 0.84] vs 0.78 [0.76; 0.82]; *P* = 0.55). Nevertheless, T2D patients showed 5% lower insulin-stimulated RQ (0.88 [0.83; 0.94] vs 0.92 [0.91; 0.99]; *P* < 0.05) and 44% lower metabolic flexibility (Fig. 3).

### Adipose tissue mitochondrial function

**Figure 4.**
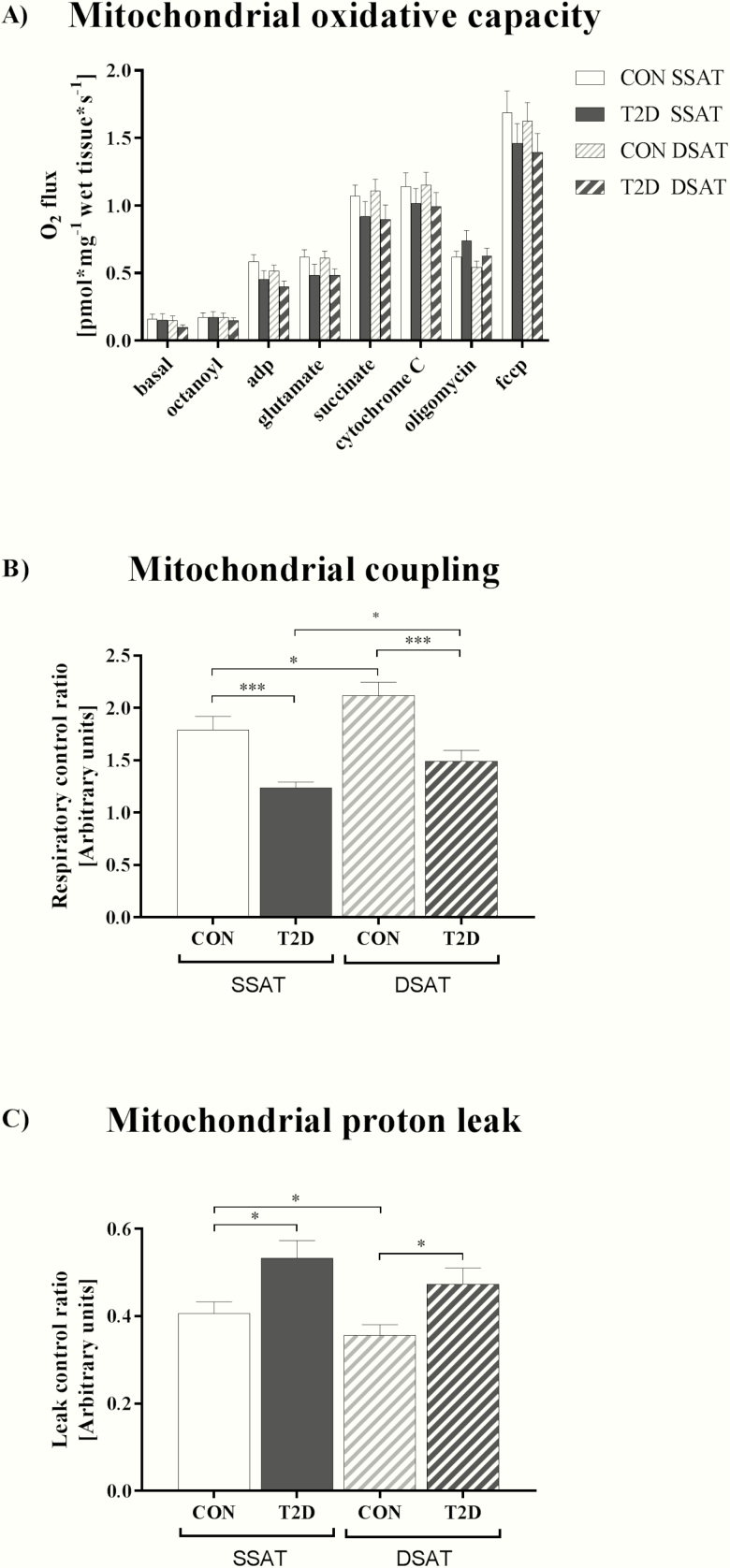
**Mitochondrial oxidative capacity (A), coupling (B) and proton leak (C) in subcutaneous adipose tissue layers.** Data are shown as mean ± SEM. * *P* < 0.05, *** *P* < 0.001, data were compared by ANCOVA adjusted for age, BMI, and total body fat and by paired Student *t*-test. All variables were assessed in n = 14 T2D patients and n = 14 CON. Abbreviations: CON, controls (individuals with normal glucose tolerance); DSAT, deep subcutaneous adipose tissue; fccp, carbonylcyanide-4-trifluoromethoxy phenylhydrazone; LCR, leak control ratio (LCR = state 4o/state u) to assess mitochondrial proton leak; RCR, respiratory control ratio (RCR = state 3/state 4o) to assess mitochondrial coupling; SSAT, superficial subcutaneous adipose tissue (SSAT); T2D, patients with type 2 diabetes.

Both groups showed similar oxidative capacity in SSAT and DSAT, regardless of whether or not it was normalized for CSA as a marker of mitochondrial content (Fig. 4A). CSA was similar in both SSAT and DSAT in T2D patients and CON and comparable between T2D patients and CON in both layers (data not shown).

In T2D and CON, SSAT featured lower mitochondrial coupling than DSAT (Fig. 4B). Accordingly, proton leak was higher in SSAT compared with DSAT in CON, but not in T2D (Fig. 4C). Interestingly, mitochondrial coupling was lower and proton leak was higher in T2D compared with CON in both SAT depots (Fig. 4B and 4C). Thus, mitochondrial efficiency is lower in both SAT compartments in T2D and also slightly reduced in SSAT compared with DSAT. Of note, adjustment for the participants’ medications did not change these results.

### Correlation analyses

**Figure 5.**
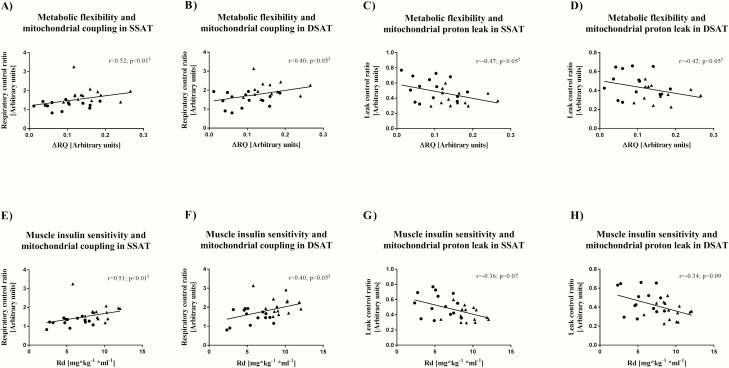
**Association of metabolic flexibility and muscle insulin sensitivity with mitochondrial coupling and proton leak in SSAT/DSAT.** Mitochondrial coupling from respiratory control ratio (RCR = state 3/state 4o respiration) and proton leak from leak control ratio (LCR = state 4o/state u) in superficial (SSAT) and deep subcutaneous adipose tissue (DSAT). Circles indicate type 2 diabetes patients (T2D) and triangles indicate individuals with normal glucose tolerance (CON). Of note, after bonferroni correction no correlation remaind significant. § indicates that the results are still significant after adjusting for age, BMI, and body fat still significant. In 1 CON participant, metabolic flexibility was not assessable, because the insulin-stimulated measurement of respiratory quotient was not performed due to technical problems. Thus, variables of metabolic flexibility were assessed in n = 14 T2D patients and n = 13 CON. All other variables were assessed in n = 14 T2D patients and n = 14 CON.

For all participants in this study, metabolic flexibility correlated positively with mitochondrial coupling (Fig. 5A and 5B) and negatively with proton leak (Fig. 5C and 5D) in both SSAT and DSAT. Metabolic flexibility correlated positively with oxidative capacity in both SAT depots (SSAT: *r =* 0.48; *P* < 0.05; DSAT: *r =* 0.52; *P* < 0.01) and negatively with DSAT thickness (*r =* −0.48; *P* < 0.05). Mitochondrial coupling correlated positively with muscle insulin sensitivity in both SSAT and DSAT (Fig. 5E and 5F). Proton leak correlated positively (SSAT: *r =* 0.51 and DSAT: *r =* 0.55, both *P* < 0.01) and oxidative capacity negatively with fasting FFA (SSAT: *r =* −0.47; *P* < 0.05; DSAT: *r =* −0.48; *P* < 0.01). Mitochondrial coupling correlated negatively with FFA during clamp in SSAT (SSAT: *r =* −0.49; *P* < 0.01). In both depots, mitochondrial coupling correlated negatively (SSAT: *r =* −0.72; *P* < 0.001; DSAT: *r =* −0.50; *P* < 0.01) and proton leak positively with HbA_1c_ levels (SSAT: *r =* 0.53; *P* < 0.01; DSAT: *r =* 0.44; *P* < 0.05). Furthermore, mitochondrial coupling in both depots correlated negatively (SSAT: *r =* −0.39; *P* < 0.01; DSAT: *r =* −0.41; *P* < 0.01) and proton leak in SSAT positively with TG levels (SSAT: *r =* 0.38; *P* < 0.01; DSAT: *r =* 0.36; *P* = 0.06). Neither mitochondrial coupling (SSAT: *r =* −0.25; *P* = 0.39; DSAT: *r =* 0.12; *P* = 0.68) nor proton leak (SSAT: *r =* 0.37; *P* = 0.24; DSAT: *r =* 0.11; *P* = 0.71) correlated with diabetes duration in both depots.

DSAT thickness correlated negatively with mitochondrial coupling in both depots (SSAT: *r =* −0.58 and DSAT: *r =* −0.50, both *P* < 0.01) and muscle insulin sensitivity (*r =* −0.59; *P* < 0.01). DSAT thickness had a positive correlation with FFA during clamp (*r =* 0.63; *P* < 0.001), TG levels (*r =* 0.48; *P* < 0.01), liver fat (*r =* 0.49; *P* < 0.01), and HbA_1c_ levels (*r =* 0.70; *P* < 0.001). DSAT thickness did not correlate with diabetes duration (*r =* −0.11; *P* = 0.71). Of note, these associations remained significant after adjusting for age, BMI, and body fat.

## Discussion

Our study shows that recently-diagnosed T2D patients with muscle and AT insulin resistance and impaired metabolic flexibility have expanded DSAT and impaired mitochondrial efficiency similarly in both SAT layers. Independently of total fat mass, muscle insulin sensitivity and metabolic flexibility associates positively with AT mitochondrial efficiency and negatively with DSAT thickness. DSAT thickness was also related to impaired suppression of lipolysis and liver fat. Thus, impaired mitochondrial function in SAT, AT insulin resistance and expansion of DSAT are defects in recent-onset T2D, which might promote muscle insulin resistance and increased substrate flux to the liver.

Impaired lipid metabolism triggers a number of negative consequences in skeletal muscle, liver, and AT itself, such as sustained excessive FFA availability, impaired insulin signaling (36, 37), oxidative stress (38, 39), and impaired mitochondrial function (40). In dysfunctional AT, inefficient fatty acid oxidation promotes ectopic lipid deposition and lipotoxicity. Fasting FFA were not elevated in our T2D patients. One possible explanation for this result is that excessive FFA are stored ectopically in T2D, as reflected by elevated liver fat content preceding the development of insulin resistance in the liver. Our cohort of recently-diagnosed T2D patients showed increased TG levels and insulin resistance in AT as assessed by the Adipo IR during fasting and during the clamp as well as by suppression of FFA during hyperinsulinemia. These results are in agreement with accepted models linking dysfunctional and insulin-resistant AT to ectopic fat deposition in recent-onset T2D (41).

Although the human white adipocyte has a small cytosolic volume with few mitochondria compared with other tissues (ie, muscle, liver), abnormal mitochondrial function in white AT may contribute to insulin resistance in peripheral tissues (16,42,43). In adipocytes, efficient mitochondrial function that results in sufficient mitochondrial ATP production is essential for balanced storage of lipid metabolites, which is related to insulin sensitivity in peripheral tissues (16). In mouse-models, impaired mitochondrial function in AT leads to systemic insulin resistance, hypertension and cardiac dysfunction (15) and may be restored by new compounds that can induce proteins relevant for mitochondrial efficiency in AT (44). A previous study showed lower expression of proteins regulating mitochondrial function in abdominal SAT of insulin-resistant compared with insulin-sensitive humans without diabetes, but these differences were less pronounced, when both groups were matched for BMI and percent body fat (14). Although the authors of this study did not provide information on the total number of humans meeting the criteria for prediabetes, some of those insulin-resistant participants had prediabetes according to their HbA_1c_ and 2-hour plasma glucose levels after a 75-g OGTT (14). Another study showed that enzyme activities, reflecting complex I and complex III activities were similar between humans with normal and impaired glucose tolerance (45). In contrast, mitochondrial function assessed by high-resolution respirometry in SAT biopsies showed controversial results in previous studies. One study reported lower mitochondrial function in obese and nonobese young patients with T2D compared with lean healthy controls (46). Another study showed similar mitochondrial oxidative capacity and coupling in patients with and without T2D following bariatric surgery-induced weight loss (47). However, these studies did not report tissue-specific insulin sensitivity, ectopic lipid deposition or depot-specific analyses in SSAT and DSAT. Additionally, they did not match the participants for body fat mass. Thus, variances in the fat mass of the participants included in the 2 studies could explain these differences. While 1 study did not report fat mass of participants (46), another showed higher fat mass in obese patients with T2D than in obese humans without T2D (47). Furthermore, both studies did not report the diabetes duration, which is an important parameter, because longstanding diabetes is associated with impaired mitochondrial function. Additionally, only 1 study excluded individuals with normal glucose tolerance with positive family history of diabetes, although multiple genes of oxidative metabolism are downregulated in healthy humans with a family history of diabetes (48). In our study, maximal oxidative capacity was not only similar in both SAT layers but also similar in patients with recently-diagnosed T2D compared with CON of similar fat mass.

The findings of a lower respiratory control ratio indicate that the electron transport system is not tightly coupled to ATP synthesis both in SSAT and DSAT in patients with T2D. Leak control ratio was higher in these patients, which suggests impaired mitochondrial integrity in both compartments. Of note, the respiratory control ratio and leak control ratio are independent of mitochondrial content and thereby allow for assessment of intrinsic mitochondrial efficiency in both SAT depots. Although mitochondrial function plays a prominent role in carbohydrate and lipid metabolism, depot-specific analyses of mitochondrial function have not been performed yet. Even though we found distinctly reduced mitochondrial efficiency in T2D compared with CON, we found only slightly lower mitochondrial efficiency in SSAT than in DSAT, which might not be of clinical significance. Previous studies indicated that SSAT and DSAT have different metabolic features, but the impairment of mitochondrial function observed in our study appeared to be equivalent in both SAT depots in T2D patients. Furthermore, mitochondrial function was also similar in both SAT depots in CON. During periods of prolonged fasting, fatty acids derived from adipose tissue serve as substrates for mitochondrial β-oxidation and ATP generation to maintain whole-body energy homeostasis (49). In turn, a sufficient amount of ATP is required for insulin signaling in adipocytes (50) and thereby crucial for insulin-stimulated antilipolytic effects (45). Development of whole-body insulin resistance was suggested to result from decreased ATP production in mitochondria for highly energy-dependent pathways in adipose tissue (10). However, segregated analyses of mitochondrial function in both SAT depots have not been performed so far. The present study observed similar mitochondrial impairments in both SAT depots in T2D and changes in both layers similarly associated with insulin resistance. Our finding that both SAT depots exhibited lower mitochondrial efficiency in T2D indicate a more general abnormality of AT energy metabolism in these patients. Furthermore, the similar negative association of mitochondrial coupling and similar positive association of proton leak in both SAT depots with HbA_1c_ may also suggest an overall contribution of mitochondrial efficiency to glucose homeostasis.

In our study, higher mitochondrial efficiency in both SAT depots related to improved metabolic flexibility. To maintain whole-body energy metabolism and homeostasis, mitochondrial function has to adapt to lipid supply, which is increased in obesity and insulin resistance (35). Similar to previous reports (8), our patients with T2D exhibit an impaired ability to switch from lipid to carbohydrate oxidation in response to insulin. Furthermore, previous data showed that ex vivo mitochondrial oxidative capacity in muscle predicts the degree of metabolic flexibility (8). Therefore, impaired mitochondrial function and plasticity in skeletal muscle might promote the lack of insulin-stimulated increase in substrate oxidation in insulin-resistant participants (8). Previously, we showed that metabolic flexibility is an independent determinant of whole-body insulin sensitivity, but is affected by AT dysfunction in recent-onset T2D patients of the GDS (11). In this cohort, metabolic flexibility associates positively with both mitochondrial oxidative capacity and efficiency in SSAT and DSAT. This indicates that not only muscle, but also SAT mitochondrial function predicts the degree of metabolic flexibility.

Despite comparable total body fat mass and abdominal SAT thickness, as assessed by ultrasound, patients with T2D feature lower SSAT and higher DSAT thickness than CON. Higher DSAT thickness in T2D patients supports findings from previous studies of a positive association between DSAT thickness and insulin resistance indices (31) and of a positive association between higher abdominal distribution of fat in DSAT and glycemic control in T2D (18, 23). Furthermore, a previous study showed that DSAT volume is associated with impaired fasting blood glucose levels in men (51). Therefore, the authors concluded that DSAT could be considered as a target for early intervention in these patients. A positive family history for T2D was shown to be associated with increased risk for T2D (52) and prediabetes (53). Interestingly, family history for T2D is not only associated with adipose tissue dysfunction, like adipocyte hypertrophy, inflammation, and fibrosis (54), but also with increased VAT and DSAT volume (55). In a tissue-specific analysis of insulin sensitivity, we found that DSAT thickness associates with circulating TG levels, muscle insulin resistance, and hepatic steatosis. Furthermore, DSAT thickness also associated negatively with metabolic flexibility and mitochondrial coupling in both SAT layers. This may indicate that higher DSAT content contributes to impaired AT energy metabolism and insulin resistance. Accordingly, the positive association of DSAT thickness with HbA_1c_ levels may indicate a contribution of this fat depot to systemic glucose homeostasis.

The present findings underline the importance of mitochondrial function in abdominal SAT for recent-onset T2D. The strengths of this study are the simultaneous analyses using gold-standard methods for assessing mitochondrial function in AT and measurements of tissue-specific insulin sensitivity in well-phenotyped individuals with T2D and well-matched CON with similar fat mass. As a gold-standard method, high-resolution respirometry allows precise measurement of AT depot-specific mitochondrial function and efficiency. As a limititation of our study, the AT samples may contain mitochondria from cell types other than adipocytes, such as stem cells, vascular endothelial cells, and smooth muscle cells. Nevertheless, these contaminations are quantitatively minor and distributed equally in all samples. However, future studies should analyse isolated mitochondria from purified adipocyte preparations. Our findings support the concept of a relevant physiological role of energy metabolism in both SAT depots for whole-body glucose homeostasis. Despite suitable statistical adjustments and drug withdrawal before our measurements, glucose-lowering therapy as well as antihypertensive and lipid-lowering medication may indirectly modulate insulin secretion and sensitivity (56) and thereby may have affected our analyses. The range of diabetes duration and the small sample size of the study group can affect the results on mitochondrial efficiency and DSAT thickness. However, in this cohort, the duration of T2D neither associated with DSAT thickness nor with mitochondrial efficiency in both SAT depots. We therefore assume that the bias introduced by the reported duration of T2D is negligible in our cohort. Furthermore, we did not assess adipocyte lipolysis directly, but indirectly via insulin-mediated suppression of lipolysis during clamp. The sample size and the cross-sectional design of the study do not allow us to draw conclusions as to a causal relationship between the abnormal mitochondrial efficiency in both SAT depots and T2D. Furthermore, the current study cannot clarify if lower mitochondrial efficiency in both SAT depots in T2D is a cause or consequence of systemic lipotoxicity and abnormal glucose homeostasis in these patients. However, recently published data challenge the latter and that study’s authors suggested obesity as the primary driver of impaired mitochondrial respiration in SAT, because mitochondrial respiration was not further deteriorated by worsening of the glycemic control (57). Moreover, mitochondrial efficiency was not associated with increased obesity (57), which corresponds to our results in humans matched for total body fat with and without diabetes.

In conclusion, mitochondrial efficiency in SAT is lower in T2D patients and associates with muscle insulin resistance and reduced metabolic flexibility independently of total fat mass. The results imply that (i) impaired mitochondrial function in SAT, (ii) AT insulin resistance, and (iii) expansion of DSAT are defects in newly-diagnosed T2D. These features might promote muscle insulin resistance and increased substrate flux to the liver. Impaired AT energy metabolism may be involved in the development of impaired glucose homeostasis and ectopic lipid deposition independently of total body fat mass.

## Abbreviations

ATP: adenosine triphosphate
AT: adipose tissue
Adipo IR: adipose tissue insulin resistance index
ANCOVA: analysis of covariance
BMI: body mass index
CON: controls (individuals with normal glucose tolerance)
CSA: citrate synthase activity
DSAT: deep subcutaneous adipose tissue
EGP: endogenous glucose production
FFA: free fatty acids
GDS: German Diabetes Study
HbA_1c_: glycated hemoglobin
HCL: hepatocellular lipid content
HDL: high-density lipoprotein
hsCRP: high-sensitivity C-reactive protein
LDL: low-density lipoprotein
MRI: magnetic resonance imaging
MRS: magnetic resonance spectroscopy
OGTT: oral glucose tolerance test
RQ: respiratory quotient
SAT: subcutaneous adipose tissue
SEM: standard error of the mean
SSAT: superficial subcutaneous adipose tissue
T2D: type 2 diabetes
TG: triglycerides
VAT: visceral adipose tissue
WSAT: whole subcutaneous adipose tissue

## Additional Information

***Disclosure Summary:*** The authors have no potential conflicts of interest relevant to this article.

***Data availability:*** To ensure data privacy of the study participants, the generated datasets of the ongoing GDS are not publicly available and are subject to national data protection laws and restrictions by the ethics committee. However, they can be requested through an individual project agreement within the GDS.
